# The experiences and needs of metastatic spinal cancer family caregivers at home: a systematic review

**DOI:** 10.1007/s00520-023-07777-5

**Published:** 2023-04-29

**Authors:** Apichat Kardosod, Judith Needham, Elisabeth Coyne

**Affiliations:** 1 School of Nursing and Midwifery, Griffith University, Logan Campus, University Dr, QLD, Meadowbrook, Australia; 2 Menzies Health Institute, Griffith University, Queensland, Australia

**Keywords:** Metastatic spinal cancer, Family caregivers, Home care, Experience and need, Systematic review

## Abstract

**Purpose:**

Family caregivers have high responsibilities for caring for persons with metastatic spinal cancer; however, understanding the experiences and needs of family caregivers face to overall recent, what is nurse-led could support them to meet their needs appropriately? Thus, the study aimed to review the experiences and needs of metastatic spinal cancer caregivers at home in the past decades.

**Methods:**

A qualitative systematic review of 8 studies was undertaken. Analysed studies were conducted in different countries (Australia, Cyprus, Italy, Kenya, Pakistan, Thailand, and Turkey), covering a population of 92 caregivers. Thematic analysis was applied to identify family caregiver experiences and needs.

**Results:**

Thematic analysis identified four key themes from the included studies: (1) complexity of needs, (2) caregivers’ role and physical needs, (3) complexity of psychosocial needs, and (4) understanding supportive care.

**Conclusions:**

The results across 8 different countries indicate that family caregivers of metastatic spinal cancer commonly face diverse challenges in many diverse geographical contexts across cultures, requiring biomedical, practical, physical, and psychosocial support from healthcare systems within the matrix of broader challenges and resources available to improve supportive care for such service users.

## Introduction

The most common metastasis from breast, lung, and prostate cancer is metastatic spinal cancer (MSC) [1]. MSC causes morbidity and disability among 80% of people who suffer from it [2]. When MSC to the spinal cord, it degrades spinal cord function, which controls various other organs in the body, leaving the person with gastrointestinal, urinary, and movement concerns [1, 3]. In addition, breathlessness, pain, loss of appetite, and fatigue are of the management of the most challenging symptoms when caregivers caring for persons with MSC need to manage and solve the problem once their loved ones have these symptoms during their palliative home care [4]. However, all symptom management experienced if their caregivers do not have any experience or care information; it is a risk for care burdensome and emotional change. The physical changes as a result of caregivers caring for persons with MSC influence the physiological, psychological, socio-economic, and spiritual well-being of patients and their families [5].

Persons with MSC are often cared for at home to allow for a positive palliative care experiences [6]. Healthcare professionals must recognise needs and support caregivers caring for persons and their family with MSC to reduce their suffering and improve their quality of life (QoL). There is a need for evidence-based care for the family in their home to improve the condition of those they care for and the psychosocial and physical health of caregivers themselves [7].

The involvement of family as caregivers is essential for persons with metastatic cancer living at home. Caregivers caring for persons with cancer who desire to care at home and often require help for activities of daily living, basic medical care, patient advocacy, and social needs [8]. These caregivers themselves require need to support so that they can be effects and maintain their own health-related quality of life while caring for persons with cancer. Other disease provide at home visits but in palliative tailored care cancer has not found that [9]. Family caring for persons with MSC at home balance activities of daily living with caring and preparedness for death at home by themselves [10].

Family caregivers face numerous dimensions of care burden, including their physical, psychological, and social health [11]. Many studies have researched family caregivers’ experiences and the need to support care at home in different country contexts [12, 13]. There are personal care challenges related to the cultures and beliefs of family caregivers of persons with metastases cancer, which affect the caring role and the health status of family caregivers and their ability to meet patient needs [14]. Nevertheless, the absolute need for high-quality supportive care for persons with metastases cancer from their family caregivers is well established among international studies [15]. Consequently, palliative care nurses have increasingly sought to understand the experiences and needs of cancer patients’ family caregivers [16].

The studies conducted in this regard have tended to be quantitative, exploring specific and predetermined variables related to the experiences and needs of family caregivers [17]. However, quantitative approaches offer limited insights into complex and multidimensional phenomena, such as psychosocial needs and perceived experiences. More observational and qualitative research is necessary to cover the multiple dimensions of family caregivers’ experiences and needs [18]. This literature review seeks to explore existing studies to understand the holistic experiences and needs of family caregivers.

Despite the apparent importance of understanding the experiences and needs of persons caring for MSC patients, there is a dearth of literature exploring this subject, but it has been identified as a key area of concern for palliative nursing [19]. Extending knowledge in this area can improve palliative care nursing to improve the health and well-being of MSC patients and their family caregivers [18]. This can improve hospital planning and policy for managing persons with MSC on their QoL at home [17]. Therefore, this study explores the experiences and needs of family caregivers caring for MSC patients in their homes based on a systematic qualitative review conducted between 9 December 2021, and 9 October 2022.

## Methods

This systematic review explores the experiences and needs of family caregivers of MSC patients in the home context [20]. The Preferred Reporting Items for Systematic Reviews and Meta-Analyses (PRISMA) was and followed and the centred review and dissemination guidelines to identify peer-review literature of sufficient data quality and sensitivity [21, 22]. This study ultimately provides a condensed and broad description of the phenomena of interest. It was registered as PROSPERO (CRD42022302504). Qualitative content analysis was used to explore the data and interpret experiences and perceptions [20].

### Search strategy

The following electronic databases were searched from January 2001 to May 2022 to identify relevant literature: CINAHL Complete, Cochrane Library, Embase, Joanna Briggs Institute EBP database, Medline, Nursing and Allied Health Database, ProQuest, Scopus, and Web of Science. However, a hand-search of relevant journals, contact with researchers, and a search of relevant grey literature sources were searched for additional studies to find all relevant studies. The keywords used in the title and abstract “experiences and needs,” “family caregivers,” and “metastatic spinal cancer,” with their respective synonyms identified from MeSH terms (“AND” and “OR”) and truncating words to include possible variations used in the literature. In addition, the search was restricted to the English language and studies of adult and elderly populations. Table 1 summarises the search strategy.

**Table 1:** Search strategy to identify studies for potential inclusion

Step	Keywords
1	famil* OR informa* OR spous* OR parent* OR unpaid* AND caregivers* OR carers* OR care giver* OR care-giver*
2	needs* OR need* OR experiences* OR health services* OR health services need* OR target population* OR perspectiv* OR liv* OR life* OR experienc*
3	2 AND 3
4	carcinoma* OR neoplasm* OR oncology* OR Neoplasm* OR Neoplasm metastases* OR vertebra* OR menispoungeal* OR spinal neoplasm* OR spinal neoplasms* OR lumbar* OR spinal* OR spinal cord*
5	3 AND 4
6	Limit in English

### Selection process

The first author (AK) screened the titles and abstracts of the initially identified articles to remove duplicates and irrelevant studies. Subsequently, the abstracts were read. Then, full texts were reviewed by AK and EC to identify articles meeting the inclusion and exclusion criteria (Table 2). Disagreement regarding study inclusion was resolved by discussion, and in cases of persistent disagreement, third (JN) reviewer consulted for final discussion. The data from the selected articles was entered into an excel table across the following headings: author, year, country, aim, method, sample, and finding (see Table 3).

**Table 2:** Inclusion and exclusion criteria

Inclusion	Exclusion
Studies of the family caregivers of MSC or metastatic bone cancer (not limited to a specific life prognosis).	Studies to reveal the experiences and needs of family caregivers that have provided in-home care for cancer.
Studies with the primary objective of revealing overall experiences and needs of family caregivers providing at-home care for spinal metastasis cancer.	Studies to explore the experiences and needs of family caregivers caring for MSC or metastatic bone cancer in a setting *outside* the home (e.g., hospital, hospice, or nursing home settings).
Primary qualitative research	Studies focused on exploring a specific aspect of the experiences and needs of family caregivers providing care for MSC (e.g., decision-making).
Published in the English language.	Family dynamics, grieving process, pain management, QoL, etc.

**Table 3:** Characteristics of the studies

Author, year, country	Aims	Methodology	Theory/framework	Theme	Sample and data collection	Finding
Demiralp et al. (2010) Turkey [19]	To describe the experiences of the family caregivers	Qualitative	Not mention	- Need palliative care at home	11 caregivers, semi-structured interview	Caregivers mentioned that caring for MSC is the anxiety about the prognosis of the malignant tumour, and the support received from significant others.
Grbich, Maddocks, and Parker (2001) Australia [29]	To explore the caregiver’s perspective, the impact of caring	Qualitative	Institutional model	- Need social communication - Need social support	20 caregivers, semi-structured interview	A home care environment is good palliative care; the nurse would be concerned with providing palliative care support for family caregivers, which is essential if existing guidelines for palliative care providers are to be met.
Lelei (2019) Kenya [27]	To identify the perceived effects of caregiving on primary caregivers	Qualitative	Thematic framework	- Psychosocial need - Uncertainly of psychological and emotional - Need barrier to home care	12 caregivers, in-depth interview	The caregiver burden was primarily due to competing tasks, lack of knowledge in managing the symptoms, difficult home-care experiences, and financial strains.
Leonidou and Giannousi (2018) Cyprus [17]	To examine metastatic cancer caregivers’ experiences within their role	Qualitative	Not mention	- Need for cancer information - Impact on caregivers - loss of control - Need social communication	17 caregivers (1 cervical metastasis), focus group interviews	Caregiving difficulties and unmet needs in caregivers with different cultural backgrounds provided further evidence for similarities and variations within caregiving experiences relating mainly to caregiver-patient relationships. Caregivers also highlighted coping and support resources that they effectively use for better adjustment to their role.
Patterson et al. (2011) Australia [26]	To identify the psychosocial needs of young people who have a parent with cancer.	Mixed method	Not mention	- Lack of preparedness - Need information on disease prognosis - Need barrier to home care	116 caregivers (8 bone and spine metastasis), focus group interviews	Caregivers have unmet needs related to understanding from friends and assistance with concentrating and staying on task.
Piazza et al. (2017) Italy [18]	To investigate caregivers’ health needs	Qualitative	Communication model	- Psychosocial needs - Physical symptoms and limitations - Impact on caregivers	25 caregivers with bone metastases, self-reported questionnaire	Caregiver-at-home care mentioned the weak points: poor physical space organization in the service, long waiting times, and limited access to healthcare providers for patients.
Sadaf Nooruddin et al. (2020) Pakistan [28]	To identify the palliative care needs of adult cancer patients from the perspective of their family members	Qualitative	No theoretical framework	- Physical symptoms and limitations - Need palliative care at home	12 caregivers (2 metastasis bone cancer), semi-structured interview	Caregivers had physical, psycho-social, religious, and financial needs. They wanted to provide context-based palliative care services to cancer patients.
Wiseso, Fongkaew, Pinyokham, and Spiers (2017) Thailand [30]	To explore the experiences of family caregivers and their perceptions of how Thai Buddhist families function in caring	Qualitative	Family system theory	- Need palliative care at home	13 caregivers, in-depth interview	Individual family members work as a unit in caregiving practices. Understanding the experiences of family members in caring for terminally ill persons with cancer can contribute to the further development of healthcare services for promoting family well-being.

### Quality assessment

The quality of included studies was assessed using Consolidated Criteria for Reporting Qualitative Research (COREQ), which has 32 questions spanning three domains. This critical appraisal tool has been widely used among researchers to analyse qualitative data [23]. Consensus was reached between the three authors using a blinded COREQ assessment process. Studies were categorised as moderate if they checked 17–24 COREQ items and poor if they checked 9–16. Three authors adjudicated study quality in the case of any disagreement on articles between the two main reviewers.

### Data analysis

The included articles were read several times to familiarise the reviewers with the methods and results. The process of qualitative thematic analysis was conducted following the guidelines of Bengtsson [24] and Elo and Kyngäs [20]. A coding framework within an excel table was developed, including data to support the developing themes. The constant comparative technique was used to identify emerging study themes and then code the group of similar phenomena into categories of inclusion articles. The themes describe the phenomena of interest and are represented within the analysis with supporting participant data from the included articles [24].

## Results

### Included articles

**Fig. 1:**
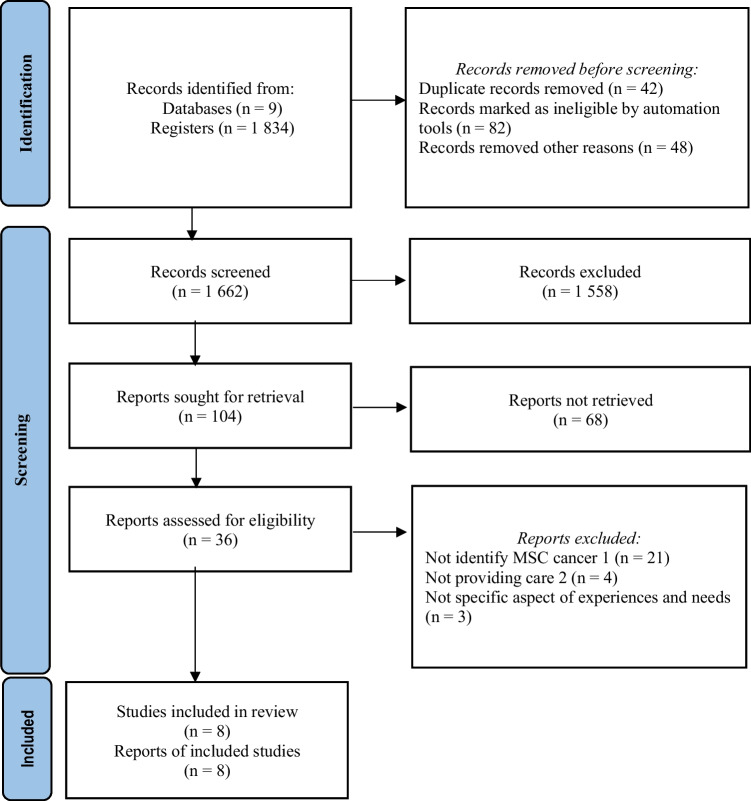
PRISMA flow diagram of the article selection process

The initial database searching resulted in 1834 articles. After eliminating duplicates and inaccessible works, the titles and abstracts of 1662 articles were examined, resulting in 36 items being identified as potentially relevant. After reading full-text versions, 8 articles were selected for the final analysis of the result. The search process is illustrated [21] in Fig. 1.

### Characteristics of included studies

The characteristics of the 8 included studies are summarised in Table 3. The studies were conducted in Australia, Cyprus, Italy, Kenya, Pakistan, Thailand, and Turkey. Collectively, they included 92 family caregivers (usually blood relatives or spouses). The studies explored the experiences and needs of family caregivers and performed data collection through individual or focus group interviews, using semi-structured or self-reported questionnaires. Three of the studies [16, 25, 26] included participants in the advanced or metastases to the bone and spinal column.

### Quality assessment results from COREQ items

The results of the COREQ evaluation of the quality are summarised to present an overall picture of the quality of the included articles [26]. Three authors critically analysed the mean score of 25.12/ 32 (SD = 2.28). The lowest score was 20, and the highest was 29/ 32; no paper complied with all 32 COREQ criteria. Criteria that over 75% of studies included interviewer/facilitator identified (item 1), the relationship established (item 6), methodological orientation (item 9), number of study participants (item 12), audio or visual record (item 19), consistency between data and finding (item 30), and clarity of significant themes (item 31). For the lower score, less than 30% of studies include: research experience and training (item 8), did the participants know about the research (item 7), research characteristics (item 8), field notes (item 20), transcript returned (item 23), and a number of data coders (item 24).

### Themes

Thematic analysis revealed four major themes pertaining to the experiences and needs of family caregivers: (1) complexity of needs, (2) caregivers role and physical needs, (3) complexity of psychosocial needs, and (4) understanding supportive care (see Table 4).

**Table 4:** Participant quotations illustrating each theme

Theme 1: Complexity of needs	
Psychosocial needs	“She feels restless due to the crowd and cannot tolerate chanting and noise. Therefore, she rarely attends social gatherings” [18, 27].
Need for cancer information	“I try to get more knowledge, to learn, to ask, to read” [17].
Lack of preparedness	“When I underwent chemotherapy, my health started failing. Every joint in my body became painful” [26].
Theme 2: Caregivers role and physical needs	
Physical symptoms and limitations	“I usually get tired and have backache” [18, 28].
Impact on caregivers	“I am trying to combine everything” [17, 18]
Loss of control	“I did not feel very well to go there when I knew that my father was in bed suffering with pain” [17].
Theme 3: Complexity of psychosocial needs	
Uncertainty of psychological and emotional	“I am very pleased to look after her” [18, 27].
Need social communication	“She asks me to empower her, I have no strength to empower her anymore” [17, 29].
Need information on diseases prognosis	“To get information about my parent’s cancer in a way that I could understand” [26, 27].
Need of palliative care at home	“I got scared and stayed back at home” [26, 28, 30].
Theme 4: Understanding supportive care	
Need social support	“My friend’s frequent visits made me very happy and so did my brother” [18, 27].
Need barrier to home care	“I do not know how long I will live. It has disturbed me a lot” [26, 27].

#### Theme 1: Complexity of needs

MSC patients’ family caregivers have a long-term, challenging experience. After knowing about the prognosis of the people with metastases cancer, especially after discharge from the hospital, many of them face the emotional challenge of coping with their loved one’s disease irreversibly approaching [16–18, 27]. However, this emotional crisis inspires them to learn about the impact of the disease and treatment options, albeit through a fraught and traumatic process. Although it is well known that curative treatment is impossible, some initially believed or hoped in the potential for supernatural intervention or that they or others could undo the damage caused by the disease and thus succeed in bringing the patient back to health [19]. Believing in alternative treatments and applying them alongside medical treatment was perceived by some family caregivers to improve the patient’s condition. While this does not mean that people with cancer can be cured, such complementary approaches may comfort patients and their family caregivers and improve their well-being [18].

Piazza, et al. [18] reported that most family caregivers became more active in learning about cancer and potential treatments during the post-discharge period while caring for patients at home. The family caregivers played a role in diagnosing and treating cancer, obtaining a sense of mastery, and practically assisting patients and healthcare providers. The involvement of caregivers should be implemented in cancer prognosis as an aspect of the whole care process.

Family caregivers were involved in holistic psychosocial and spiritual aspects of care which become increasingly important during the palliative phase of MSC care, such as coordinating the religious needs of patients. For instance, Sadaf Nooruddin et al. [28] reported that the frequency of prayer increased among patients and caregivers. Expectations about what will happen, haunt and worry them constantly:

Now, I recite the Holy Quran and offer prayers more frequently. [28]

Others were worried about the future of their illness and do not know how the disease will progress. Their apprehensions constantly trouble them [18, 29], as expressed by family caregivers:

I am really worried whether the tumour is totally removed or not, if it will recur or not, and if my son will be able to walk again. [29]

They always find more information about the prognosis and care treatment at home [16, 28]. In this situation, the exacerbations of the illness increase the family caregivers’ anxiety and aggravate their sense of caring and uncertainty because people with metastases cancer prognosis generally have unstable conditions [18], as expressed by caregivers:

I am afraid that the tumour will disseminate, and I am worried about losing her, and I try to get rid of that idea. [18]

These circumstances may cause the family caregivers to feel that they do not have a sense of control over their own lives, which subsequently complicates their continued caring abilities [17]. In addition, some of the family caregivers experienced feeling guilt and loneliness while caring for their persons with metastases cancer, and their increased needs for religiosity while caring at home represented “an attempt to capture a broad range of needs across the cancer journey” [16].

#### Theme 2: Caregivers role and physical needs

Family caregivers caring for persons with spinal metastasis cancer at home have been observed to carry an enormous physical and emotional burden from their care role. The malignant tumour effects result in suffering in activities of daily living and anxiety for caregivers as well as patients themselves [18, 29]. Family caregivers commonly experience exhaustion and physical problems associated with the physical labour of caring, such as practical difficulties in assisting patients with activities of daily living, overlooking their own personal health problems, poor management of work-life burdens, social burdens, and overlooking their parental roles and other relationships in their lives [16, 27]. Family caregivers’ burden is commonly described in terms of the exhausting demands of care:

Usually, I take him up from the bed and then put him back to bed again. I usually get tired and have backache. [18]

The emotional burden could be instrumental in physical problems, but both are often exacerbated by a lack of sleep when persons with metastases cancer come to the hospital for follow-up treatment. This can also be associated with loss of appetite and complete exhaustion, resulting in a highly emotional situation:

I have been exhausted since I have come to the hospital. I do not feel hungry, I cannot eat. [18]

These experiences are associated with significant physical needs among family caregivers of persons with MSC. In the case of the caring process, the family caregivers face the physical limitations of the diseases and treatment due to caring at home. In this situation, the family caregivers compromise their daily life activities, and weakness, body aches, and other pains and illnesses affect their ability to undertake self-care, resulting in feeling anger as well as exhaustion, and a lack of interest in undertaking their own activities of daily living:

I do not feel like doing anything [household chores and work]. I feel very weak. I used to work a lot before, but now I cannot do so. My body aches a lot, and I cannot walk. [26]

Thus, the caring experience results in a massive physical and psychological burden for family caregivers. They struggle to manage work-life balance; family caregivers’ difficulties in the work domain include more days off work, leaving their jobs temporarily, or switching from highly demanding job positions to part-time or less responsible (and less well-paid) roles, in order to prioritise caregiving [16]. This results in the feeling of being overwhelmed in trying to juggle roles and a reaction of withdrawal into themselves, abandoning their own social lives:

I am trying to combine everything and I hold them (my thoughts and feelings) for myself. [16]

#### Theme 3: Complexity of psychosocial needs

Family caregivers often talk about various negative, positive, constructive, and psychosocial needs they experience while caring for MSC patients at home. As the illness progresses, patients have an increasing dependency and need more attention. Thus, the tendency to think vivaciously and constructively becomes continually more significant [17]. Family caregivers’ experiences engendered several positive emotions, including confidence, belonging, honour, and appreciation. They expressed an increase in spiritual presence in their lives and family relationships. Experiencing positive thinking made family caregivers easier, and generally gave them renewed hope:

Looking after her, being with her is a very nice feeling. [18]

They often reflected that they used cognitive and emotional strategies to cope with daily difficulties and distress stemming from the family caregivers’ role:

If he has more years to live, then he will live if not … I owe to take care of him as far as I can. [16]

Similarly, the emotion of coping constructively and reflecting that the situation might have been worse was found to be helpful in dealing with stressful experiences, relating to spirituality and hope:

God willing, there will be no need to something else after the operation, my God helps to all patients, I want my son get over, we have the hope, and he will get over. [18]

However, some family caregivers felt that they failed in providing care and that they did not have enough informational support to enable them to function effectively. As described above, such caregivers were more likely to leave their other interests and occupations and even wholly abandon their social lives. In such circumstances, with a restricted scope of holistic life activities, they were more prone to negative psychological emotions, a lack of willingness to seek help from others due to social withdrawal, and a more negative fatalistic orientation in conceptualising the MSC patient’s condition:

I am trapped in worry, anxiety and fear. [16]

Negative or positive feelings tend to affect the performance of family caregivers in terms of their care provision, their personal and professional roles, and their psychosocial needs related to fear, anxiety, and social isolation. Experiences with fear and anxiety of the disease and its treatment relate to concern and uncertainty about the future, and they consequently perceived that they needed information to support them:

At first, I thought that I would die and if I survived, my children would leave me due to this disease [misconception about disease being contagious]. I thought that I would faint or something bad would happen to me. [26]

#### Theme 4: Understanding supportive care

Family caregivers experienced appreciation of supportive care they received from other family members, neighbourhood figures, and healthcare service providers, which lifted their spirits and positively affected their coping and acceptance of MSC and themselves. Concerning informational support, family caregivers affirmed that having good relationships with others was vital in caring for MSC patients, with encouragement from supporters giving them emotional strength. They said that in this way, they felt that they could express their feelings:

My friend’s frequent visits made me very happy, and so did my brother. [18]

This support led them to be more accessible and take time for themselves. However, having a broad social network that supports them is essential, especially when family support is not permanent, because it may mitigate their isolation. Consequently, this network allows them to share experiences with people who are not sentimentally involved in the experience of the illness per se. Despite its importance, many caregivers experienced a lack of availability and help from their family caregivers, friends, neighbourhoods, and communities. However, the most fundamental challenges family caregivers faced was their need for financial support, because diagnosis and treatment were costly, which exacerbated the burden of emotional distress with financial stress:

There is no fun in life anymore. All I think about is cancer. Maybe this will happen, or that will occur. I do not know how long I will live. It has disturbed me a lot. [26]

Many family caregivers reported that they experienced hardship in managing their finances, and they found emotional support from other people who helped and interacted with them [29, 30].

## Discussion

This systematic review describes the experiences and needs of family caregivers caring for MSC patients in their homes in terms of personal dimensions. The literature review demonstrated that these experiences and needs involve aspects concerning the acceptance of the advanced cancer process and uncertainties about the prognosis of the symptom for persons with MSC in the future [16, 27, 28, 31]. Although the experiences and needs identified in this review cannot be considered universally generalisable, there is some degree of representativeness as the analysed studies were conducted in five different countries with very different cultural contexts. Similarly, it is notable that although the experiences and needs of each family caregiver are unique in their traditional and cultural contexts, and not all share every experience and need, the experiences and needs of caring for family caregivers at the metastasis stage of cancer comprised the same everyday experiences and needs.

This review identified the experiences of the family caregivers, learning about the impact of a cancer diagnosis on persons with MSC and their carers, treatment roles, and religious, physical, emotional, and financial needs. Caregivers learned and implemented the information they received about treatments to decide what interventions they considered best for patients [32]. Given the gravity of such issues, many family caregivers feel overwhelmed by the situation, reflecting on their decisions and emotional burdens throughout the illness. Hence, this is imperative that palliative care nurses understand and be mindful of the impact of such situations on the personal lives and psychological resilience of family caregivers. It is necessary to give caregivers as much support and information as possible to find meaning and achieve mastery in terms of the experiences and needs they are meeting and living with for patients whilst improving their health and well-being. Aside from the healthcare responsibility to family caregivers, their improved physical and psychosocial health will translate into improved quality of care and patient outcomes, which can be accentuated by feeling relieved and supported by healthcare professionals [33].

This review explored the emotional and physical burdens faced by family caregivers and how caring tends to negatively affect their QoL during caring for persons with MSC at home, which must be a significant concern for palliative nursing [34]. Nurses must promote the development of policies related to family caregivers’ role in palliative care at home, which ought to be objective to improve the recognition of the family caregivers and knowledge for caring for their needs [35]. In the same situation, almost all participants faced financial challenges. Healthcare financial systems across the country could be attributed to health insurance to the citizen’s coverage by any public health, insurance, and financial system for cancer treatment. In the broader context, family caregivers for cancer patients should be supported by work policies to protect individuals’ work positions, retirement rights, and access to education and training [33].

This review found that family caregivers have a wide variety of holistic needs, including spiritual needs, particularly salient in oncology cases and palliative care [35]. The absence of psychosocial and spiritual sources of support for family caregivers may leave them and the field of oncology without some of the support mechanisms specifically necessary for the palliative care of persons with MSC. A dearth of such support can result in patients and caregivers being cut off from many resources, particularly social interactions, which worsens their pre-existing problems and leads to adverse emotional and physical health outcomes. Hence, the association between family caregivers and the type of oncology people should be considered a central focus in care support needs.

In this setting, the objective of enabling sustainable experiences and needs and helping family caregivers maintain communication and information that allows them to speak freely about their aspects of these experiences and needs has been highlighted. Specific features may have positive repercussions in the aspects, such as shared decision-making, in which frank and sincere dialogues have been observed to lead to cooperation [36]. Promoting and articulating experiences and needs could allow caregiver relationships with others to remain intact and promote greater closeness and psychosocial resilience [16, 29].

This review emphasises the importance of the experiences and needs of informal and formal support for family caregivers. It is necessary to promote and develop strategies that facilitate adequate family caregiving and make resources available that help these people in their daily lives (when considered necessary). This review challenges the current socio-cultural care model and emphasises the importance of the need to modify its objectives and limits. The experiences and needs of oncology nurses as well as family caregivers must be assessed, and informal support systems for the latter should be identified. Future research should explore the nature of such sources of care support and responsibilities, encouraging greater social and cultural participation, which may intersect with community and faith groups in some socio-cultural contexts [37, 38]. These changes can produce the best possible conditions for family caregivers, enabling them to obtain adequate socio-cultural support in every situation [39]. For this work, the responsibility of professionals and the population, in general, must be increased, developing a culture change and awareness of this need [40]. Nursing professionals should promote this social support and ensure their accessibility and continuity of care, to sustain oncology and palliative care at home [41].

On the other hand, information about supportive activities of daily life and self-care services from the review underscores the need for health professionals to improve their communication with the family caregivers and their support [42]. In this respect, nurses play a crucial role in providing instructions about how to care for the persons with MSC and information about treatment, medications, and the availability of socio-cultural health assistance for care support. These indications have been observed to have positive repercussions on the well-being of caregivers, helping to lighten the care burden, uncertainty, and loneliness they experience. Similarly, this experience is the need of their family caregivers from a multidisciplinary team. Additionally, they feel valued and recognised as team members when adequately presented information [43]. Therefore, the health care team’s objective must be that the family caregivers themselves are also included in care because they frequently experience a lack of emotional and psychological support and feel that their needs are not valued. Family caregivers must be supported to accept their situation, maintain care satisfaction and control, and reduce their fear and insecurity when this support is given [44]. In addition, caregivers were encouraged to care based on the factors associated with family caregivers’ experiences and needs for engagement in caring, including relationship quality, perceived caregivers to care, relationship status, number of care hours delivered, and maternal status would be continuous in the family that to meet their needs.

The reviewed studies all had appropriate designs for the research questions posed, described in detail the sampling strategies used, and provided comprehensive information regarding the collection and analysis of the data supplied by the participants. However, a gap was found regarding the researchers describing their beliefs and possible influences on the interpretation of study data, particularly when conducting interviews and data analysis. Researchers should address it in their studies because it is crucial to assess the confirmability of the research [45].

The main limitation of this review is that few relevant articles were found, and they were mainly German to Australian and European socio-cultural contexts. However, the studies were conducted in eight countries with different cultures. Although this cultural difference could be viewed as limited, it could also be considered a strength because the result between the different cultures does not differ substantially. By combining the insights from the sampled countries, the validity of the findings of each particular study is increased. The selection of the articles was rigorous, using specific inclusion and exclusion criteria verified by various authors. Two reviewers conducted qualitative content analysis bestows quality and rigor in the study. This study’s findings regarding the experiences and needs of caring for family caregivers with MSC and bone metastases are beneficial for palliative care nurses and other professionals involved at the end-of-life care in home settings, helping develop ways to provide supportive care to meet service user needs.

## Conclusion

The number of persons with MSC increases with every type of cancer. Providing the biomedical needs of patients are met, it is also generally preferred for health system efficiency to facilitate palliative care home where possible (and desired by patients), freeing up clinical resources in hospitals for other patients. Under such circumstances, the needs of most palliative oncology patients are met by family caregivers, who undertake substantial responsibilities to assist patients with activities of daily living as well as biomedical care assistance in the home. Therefore, growing interest has emerged in understanding the experiences and needs of these family caregivers and what it means for care and their lives [32].

Providing care for family caregivers with MSC at home has a significant impact on all family members of persons with cancer and the broader personal, social, and professional spheres of life for family caregivers. Being involved in dying entails a prodigious physical and psychological effort, together with a substantial limitation in the normal development of their own lives. Additionally, the family caregivers must face and establish a new relationship with patients with a new ethos, all without the help of a formal structure providing necessary support. In light of the data, it is necessary to promote and generate more research into the phenomenon, both from a holistic perspective and by probing each aspect.

Palliative nursing care must be more responsive to the expectations and needs of family caregivers who care for persons with MSC in their homes. Family caregivers at home who care for persons with MSC must be a priority of palliative care nursing, navigating changing care needs in socio-cultural contexts to provide modern and holistic healthcare services.

## Recommendations

Training video-assisted communication strategies for difficult conversations and supporting persons at home. There is insufficient evidence regarding appreciating any benefit of access to palliative care [46]. Future work is needed to evaluate the use of video-assistant in palliative care, and improve design could be effective for caregivers caring at home to meet their needs.

Future research with caregivers how to capture this ethnography needs an assessment of understanding how caregivers is done at home. Studies of ways to achieve this must refocus attention on family caregivers and advance the research and development of the new knowledge that nurses require to attend to service users’ holistic healthcare needs for palliative care at home.

Building clinical practices inclusive of the family providing care at home [47]. Palliative care follows up telehealth or phone calls, and explicitly examining its use of caregivers’ specialist palliative care provision is recommended. These could affect caregivers caring for persons with cancer at home and help meet their needs.
